# Acceptability and Feasibility of Real-Time Antiretroviral Therapy Adherence Interventions in Rural Uganda: Mixed-Method Pilot Randomized Controlled Trial

**DOI:** 10.2196/mhealth.9031

**Published:** 2018-05-17

**Authors:** Angella Musiimenta, Esther C Atukunda, Wilson Tumuhimbise, Emily E Pisarski, Melanie Tam, Monique A Wyatt, Norma C Ware, Jessica E Haberer

**Affiliations:** ^1^ Mbarara University of Science and Technology Mbarara Uganda; ^2^ Harvard Medical School Boston, MA United States; ^3^ Wake Forest School of Medicine Winston-Salem, NC United States; ^4^ Harvard Global Cambridge, MA United States; ^5^ Harvard Medical School and Massachusetts General Hospital Boston, MA United States

**Keywords:** real-time adherence monitoring, SMS, mobile health technologies, antiretroviral therapy, acceptability, feasibility study

## Abstract

**Background:**

Wireless electronic adherence monitors can detect antiretroviral therapy (ART) adherence lapses and trigger interventions in real time, thus potentially avoiding unnecessary HIV viremia. Evidence about the acceptability and feasibility of these monitors and associated interventions, however, is limited.

**Objective:**

The aim of this study was to assess the acceptability and feasibility of real-time adherence monitoring linked to text messaging (short message service, SMS) reminders and notifications to support adherence among individuals living with HIV who are taking ART in rural southwestern Uganda.

**Methods:**

Individuals living with HIV who were initiating ART were enrolled in a pilot randomized controlled trial and followed up for 9 months. Participants received a real-time adherence monitor and were randomized to one of the following study arms: (1) scheduled SMS, (2) SMS triggered by missed or delayed doses, or (3) no SMS. SMS notifications were also sent to 45 patient-identified social supporters for sustained adherence lapses in the scheduled SMS and triggered SMS arms. Study participants and social supporters participated in qualitative semistructured in-depth interviews on acceptability and feasibility of this technology. An inductive, content analytic approach, framed by the unified theory of acceptance and use of technology model, was used to analyze qualitative data. Quantitative feasibility data, including device functionality and SMS tracking data, were recorded based upon device metrics collected electronically and summarized descriptively.

**Results:**

A total of 63 participants participated in the study. Participants reported that real-time monitoring intervention linked to SMS reminders and notifications are generally acceptable; the predominant feedback was perceived utility—the intervention was beneficial in motivating and reminding patients to take medication, as well as enabling provision of social support.

The intervention was found to be technically feasible, as data were obtained from most participants as expected most of the time. Potential challenges included the impact of the technology on confidentiality, shared phone ownership, usability skills, and availability of electricity.

**Conclusions:**

Real-time adherence monitoring integrated with SMS reminders and social support notifications is a generally acceptable (based primarily on perceived utility) and feasible intervention in a resource-limited country. Future efforts should focus on optimized device design, user training to overcome the challenges we encountered, cost effectiveness studies, as well as studying the monitoring aspect of the device without accompanying interventions.

**Trial Registration:**

ClinicalTrials.gov NCT01957865; https://clinicaltrials.gov/ct2/show/NCT01957865 (Archived by WebCite at http://www.webcitation.org/6zFiDlXDa)

## Introduction

### Adherence to Antiretroviral Therapy

HIV or AIDS remains one of the biggest public health challenges, especially in developing countries, which accounts for over 70% of the 36.9 million people living with HIV or AIDS (PLWHA) globally. Antiretroviral therapy (ART) adherence is critical for achieving viral suppression, which leads to improved clinical outcomes and reduced secondary transmission. Despite simplification of HIV treatment (eg, single tablet and once daily dosing regimens) and improved access to ART, adherence remains challenging [1]. Nonadherence can result in HIV viremia, ART failure, and drug resistance, which can lead to deaths because of limited or complete inaccessibility of alternative therapies in resource-limited countries. Traditional approaches to adherence monitoring (eg, self-report, pill counts, and pharmacy refills) do not enable real-time interventions, as they may not detect nonadherence until viral suppression has been lost [2].

### Potentials of mHealth Technologies

mHealth technologies can potentially improve adherence to long-term medications through real-time medication and pill refill reminders, prompting social support and enabling medication monitoring [3]. Real-time wireless adherence monitors, for example, can detect adherence lapses as they occur, and interventions such as SMS reminders can be instituted before the loss of viral suppression [4]. Widespread cell phone ownership and mobile network coverage in sub-Saharan Africa provide a promising platform for the implementation of mobile-based interventions, which can help overcome structural barriers such as transportation to a clinic and limited human resources and enable frequent intervention when and where it is needed [5,6].

SMS reminders unlinked to real-time adherence monitoring have been shown to improve adherence to ART in resource-limited settings [7-9]. The use of these SMS reminders has been reported as acceptable in Uganda [7], Kenya [8], South Africa [9,10], India [11] Brazil [12], and the United States [13]. Data, however, have been more mixed in China [2,14]. Weekly and twice-weekly SMS text message (short message service, SMS) reminders have been reported to increase adherence in Kenya and Nigeria [8,15]; however, no benefit was seen with daily SMS text messages in another study in Kenya [16]. SMS reminders triggered by lapses in real-time adherence monitors have been shown to improve adherence and reduce lapses in adherence in several but not all settings [2,11,12,17-20]. Compared with standard electronic monitoring, real-time electronic adherence monitoring (using Medication Event Monitoring System—MEMS) plus home visits for sustained interruptions increased average adherence in Uganda [21].

### Acceptability and Feasibility of mHealth Technologies

The acceptability and feasibility of SMS reminders in Uganda, however, have not been well studied. SMS reminders triggered by late or missed doses detected by real-time adherence monitors improved overall antiretroviral adherence in China [18] but did not significantly improve adherence in South Africa, although it had fewer sustained adherence lapses [19].

Given the promise of real-time adherence monitoring and mobile-based interventions, the variability in effectiveness, and scarcity of literature, more thorough assessments of their acceptability and feasibility are needed. We conducted a pilot randomized controlled trial (RCT) based on real-time intervention linked to SMS reminders and notifications to support adherence among PLWHA taking ART in rural southwestern Uganda. As previously published [17] (Trial ID number: NCT01957865), adherence significantly improved for participants receiving scheduled SMS reminders that were sent daily and then weekly.

Moreover, participants reported that the intervention encouraged medication adherence through feeling cared about, habit formation, and a desire to show commitment to taking their medication [22]. Within the context of this pilot RCT, we used both qualitative and quantitative methods to assess the acceptability and feasibility of the intervention (a package of real-time adherence monitoring, SMS reminders for patients, and SMS notifications for social supporters).

## Methods

### Ethical Review

Ethical approvals for this study were obtained from the Institutional Review Committee of Mbarara University of Science and Technology, the Uganda National Council for Science and Technology, and the Partners Human Research Committee at Massachusetts General Hospital. Participants provided signed informed consent before study participation. All participants’ data were securely stored electronically and protected by passwords. As a cultural practice in Uganda, participants were given 10,000 Ugandan Shillings (per trip; equivalent of approximately US $4) to cover transportation costs if they came to the research offices for an interview).

### Study Site and Participants

This study involved two types of participants: PLWHA (called study participants) and their social supporters. Study participants initiating ART were recruited from the Immune Suppression Syndrome Clinic at Mbarara Regional Referral Hospital (MRRH), a rural public hospital that dispenses free ART to over 10,000 people living with HIV in southwestern Uganda. We focused on ART initiators because they are not yet accustomed to taking medication; intervening at this level could potentially result in developing medication adherence habits. HIV status was identified by checking participants’ medical records Recruitment criteria for study participants are shown in Textbox 1.

Each study participant named one to two social supporters who met the following criteria, as shown in Textbox 2.

### Study Procedures

All study participants received a real-time adherence monitor (Wisepill Technologies, Cape Town, South Africa; see below) and training on its function and use (eg, filling and removing antiretroviral medications and device charging). Before enrollment in the study, potential participants were assessed for adequate cellular reception in their homes on a network supported by the technology used in this study (MTN or Airtel). Participants were given solar chargers and sent an SMS to charge the monitor as needed. A simple random number generator was used to determine study arm assignments. After screening and consenting, participants were randomized 1:1:1 as follows:

1. Scheduled SMS (also known as SMS reminders) plus real-time adherence monitoring (scheduled SMS arm)—Study participants received an SMS reminder daily for 1 month, then weekly for 2 months. For the next 6 months, study participants received an SMS only if no signal was received from the monitor within 2 hours of the expected dosing time, and an SMS notification was sent to one to two social supporters if no signal was received for more than 48 hours.
2. Triggered SMS (also known as SMS reminders) plus real-time adherence monitoring (triggered SMS arm)—For the entire 9-month study period, study participants received an SMS only if no signal was received from the monitor within 2 hours of the expected dosing time. For the latter 6 of the 9 months, an SMS notification was sent to one to two social supporters if no signal was received for >48 hours.
3. Real-time adherence monitoring only (called the control)—Study participants in this arm received no SMS reminders.

Literature about the appropriate frequency of SMS texts is mixed up. For example, weekly SMS reminders increased adherence in Kenya [16], whereas no benefit was observed in Cameroon [23]. We therefore used different types of SMS texts as we sought to test SMS communication to patients and social supporters sequentially to efficiently study which ones were most feasible, acceptable, and impactful. For instance, our findings published elsewhere indicate that unlike SMS linked to late or missed doses, scheduled SMS significantly increased adherence [17] and were preferred to linked SMS texts [22].

Social supporters were identified by study participants at enrollment. Social supporters were enrolled into the study at month 3 and were contacted during week 2 before sending them SMS notifications to ensure ongoing relationships with study participants when the SMS notifications began. They were sent SMS notifications during months 4 and 9 after potential lapses in adherence of study participants had been identified. Social supporters were not given specific instructions on the kind of support to be provided but were generally encouraged to support study participants.

**Recruitment criteria for study participants.**

- Age ≥18 years
- Personal cell phone ownership
- Ability to read short message service (SMS) messages
- Availability of mobile network at participants’ homes
- Willingness to receive SMS reminders
- Ability and willingness to provide informed consent
- Living within 20 km from Mbarara Regional Referral Hospital (to facilitate participant follow-up)
- Ability to identify at least one social supporter to join the study

**Inclusion criteria for social supporters.**

- Age ≥18 years
- Ongoing relationships
- Cell phone ownership
- Knowledge of the study participant’s HIV status
- Willingness to provide informed consent
- History of providing social support (eg, assistance to travel to the clinic and medication adherence advice) to the study participant

### The Intervention Technology: Real-Time Adherence Monitor (Wisepill Device)

Dimagi (a mobile technology solutions company, Cambridge, Massachusetts, United States) and Yo! Voice Solutions (a gateway service provider, Kampala, Uganda) developed the SMS reminder system that was hosted in the open source application, CommCare. This application was then linked to the real-time adherence monitoring system.

The content of SMS reminders was customized and determined by each participant to reduce the risk of unintended HIV status disclosure. Notifications were also customized on request. The default message was “This is your reminder.”

**Figure 1:**
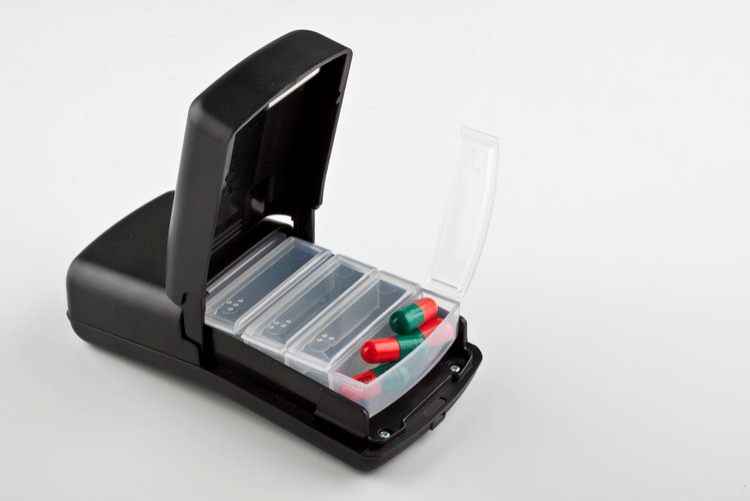
The Wisepill device.

Powered by a rechargeable battery, the real-time adherence monitor (Figure 1) is a medication container that can hold up to sixty small pills. When an individual opens it to take pills, the device records a date-and-time stamp. An internal modem and subscriber identity module card enable the device to send a real-time mobile signal to a secure Web server (hosted in South Africa) by General Packet Radio Service (GPRS). Receipt of this signal was taken as a proxy for taking medication. GPRS maintains the data in transit until acknowledgment of receipt by the Web server, which minimizes possible data loss because of power failure or lack of Internet connectivity. Data transmission is backed up by the SMS to mitigate possible temporal GPRS network disconnections. In the event of inadequate mobile network coverage, the monitor stores openings in flash memory and sends them when the network becomes available. The monitor also transmits a daily heart beat that indicates current battery life, remaining airtime balance, and signal strength as indication of its functionality. The monitor can be charged using electricity or a solar device. Its battery life was 3 months at the time of the study but has since been improved to 6 months.

### Data Collection

Study participants were seen at baseline, 3 months, and 9 months for collection of socio-behavioral data and viral load assessment. Signals sent after opening the real-time adherence monitor to the study server comprised the adherence data.

Semistructured qualitative interviews were conducted after month 3 (known as interview 1) and after the first 48-hour lapse (known as interview 2), or at study exit if there was no such lapse (also known as interview 2), reflecting two planned interviews per participant. In-depth semistructured interviews with a purposeful sample of social supporters were conducted within 2 weeks of a lapse by their respective study participant. Their selection was based on the study participant’s explanation for the lapse, social support characteristics, and variations in the types of social support provided. Closed and open-ended questions were asked of social supporters at exit exploring various aspects such as challenges and experiences to social support and understanding of and responses to the intervention SMS notifications and the type of voluntary and requested help or support presently given to the study participant toward adherence.

Research assistants who were bilingual in English and the local language (Runyankole) and trained in qualitative research and research ethics carried out semistructured in-depth interviews at the research office, participants’ homes, or any other place preferred by the participants. Interview topics with study participants covered the following: (1) preferences for content; frequency and timing of SMS reminders, (2) understandings and experiences of SMS reminders, and (3) understandings and experiences of real-time adherence monitoring. Social supporter interviews explored the following topics: (1) selection of social supporter by the study participant, (2) type of social support given, and (3) likes and dislikes of the SMS notification. All questions in the interview guide were translated into the local language (Runyankole) and back translated to English by a different translator. Interviews were conducted in the local language, digitally recorded, and translated to English during transcription for analysis. Following each interview, the research investigators reviewed transcripts for quality, clarity, and detail.

Quantitative effects of the intervention on adherence, qualitative interpretation of the mechanisms for intervention effects, and exploration of the social supporter aspect of the intervention are reported elsewhere [17,22,24].

Feasibility data were obtained by recording the number of adherence monitors reported or detected to malfunction, percentage of functional adherence monitors at the end of the study, number of battery failures or changes, percentage of data lost because of technical issues, and number of SMSs not sent as planned. Some qualitative aspects of feasibility were also explored, including sources for storing ART other than the monitor, use of the monitor to store other medications, and monitor openings for reasons other than pill-taking.

### Data Analysis

The unified theory of acceptance and use of technology (UTAUT) model, which has been shown to predict a substantial portion of the acceptance of health information technology, served as the conceptual framework for this analysis [25]. In this model, technology adoption is influenced by four major constructs as perceived by an individual user: (1) performance expectancy or perceived usefulness, (2) effort expectancy or percieved ease of use, (3) social norms (ie, how others perceive the individual’s use of the intervention), and (4) facilitating conditions (ie, the availability of technical and organizational infrastructure to support use of the intervention). We used an inductive, content analytic approach to analyze the qualitative data [26]. For this paper, we used the qualitative data management computer software program NVIVO 10 (QSR International., Melbourne, Australia) to organize the data.

With substantial input from JEH, NCW, TW, and MAW, AM reviewed transcripts for content relevant to acceptability drawing from the UTAUT model; developed a coding scheme based on the content identified; coded the data; sorted and reviewed the coded data to develop descriptive categories; and mapped the descriptive categories onto the domains of the UTAUT model (focusing on perceived usefulness, perceived ease of use, social norms, and facilitating conditions). Illustrative citations were then selected from the coded data. Quantitative data about the feasibility of the intervention were recorded and summarized descriptively using STATA 13 (StataCorp., College Station, Texas, USA).

## Results

### Participant Characteristics

Of 195 screened individuals, 63 were enrolled in the study from September 2013 to October 2014, whose 9-month follow-up ended in June 2015. One participant was later discovered to be HIV negative and was excluded from the analysis. The criteria for excluding the rest is indicated in Textbox 3 (participants could have >1 criterion). Table 1 indicates the participants’ characteristics.

We had 63 participants initially; scheduled SMS arm (21 participants), triggered SMS arm (21 participants), and control arm (21 participants). One participant was found to be HIV negative after randomization and disenrolled from the triggered arm. Four study participants were lost to follow-up (2 in the triggered SMS arm, 2 in the control). A total of 41 social supporters completed the study. One social supporter died, one was lost to follow-up, and two were disenrolled per the study participants’ requests.

**Exclusion criteria for study participants.**

- Living more than 20 km from Mbarara Regional Referral Hospital: 111 (56.9%, 111/195)
- Having no personal cell phone: 72 (36.9%, 72/195)
- Unwillingness or inability to name at least one social supporter: 29 (14.9%, 29/195)
- Unwillingness to have mobile reception tested at home: 10 (5.1%, 10/195)
- Inadequate mobile network reception: 4 (2.0%, 4/195)
- Inability to provide informed consent: 4 (2.0%, 4/195)
- Aged <18 years: 2 (1.0%, 2/195)

**Table 1:** Participant characteristics.

Characteristic	Statistic
Participants included in the study, n	63
Participants who completed the study, n (%)	58 (92)
Had electricity in their homes, n (%)	38 (65)
Females, n (%)	41 (65)
Able to read and write, n (%)	61 (97)
Median age in years	30
Median follow-up time in months	8.9

**Figure 2:**
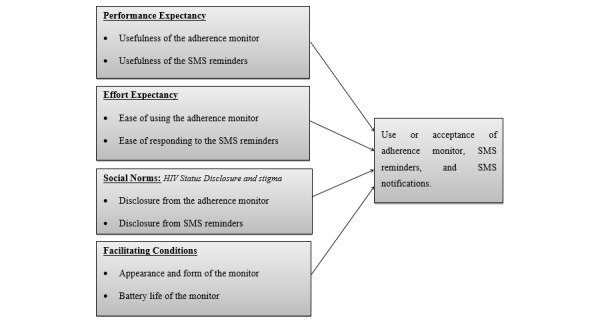
Organization of qualitative data on acceptability following the unified theory of acceptance and use of technology (UTAUT) model. SMS: short message service.

Interviews were carried out as follows: (1) semistructured interviews were conducted after month 3 with 41 study participants of the scheduled SMS arm participants (21 participants) and the triggered SMS arm (20 participants) just before completing the first intervention, before switching to the second type of intervention (known as interview 1); (2) semistructured interviews were conducted after the first 48-hour lapse with 30 study participants drawn from the 41 participants of the scheduled SMS and triggered SMS arms (known as interview 2); (3) semistructured interviews were conducted at study exit with 30 participants if there was no lapse (also known as interview 2)—these included 11 participants from scheduled SMS arm and triggered SMS arm that never lapsed and 19 participants from the “control” arm; and (4) semistructured interviews were carried out with 10 social supporters.

### Intervention Acceptability

Acceptability results are presented following the UTAUT model (Figure 2) and detail the performance expectations, effort expectancy, social norms, and facilitating conditions associated with the three components of the intervention: the real-time adherence monitor, participants’ SMS reminders, and SMS notifications for social supporters.

#### Performance Expectancy or Perceived Usefulness

Study participants found the electronic monitoring device useful, especially in supporting their pill-taking behavior. They described feeling motivated by the real-time monitoring because they interpreted monitoring as meaning study staff “cared” about them. Accordingly, study participants did not want to be “caught” not adhering to their pills; they felt that such behavior could constrain their ongoing relationships with the study staff and “disappoint” them:

I know that every time I open it [device], that light that flashes by its side indicated that a message has been sent to you people telling you that I have opened and taken my pills. I feel it with in my heart not to disappoint you people so I try to open it on time, whenever I can.

Triggered SMS arm, male, study participant

It [device] reminds me and keeps well my pills I make sure I open it to take my pills because I don’t want you to catch me...I mean I don’t want you people to know that I don’t take my pills and I get problems with you.

Scheduled SMS arm, female, study participant

Participants found the SMS reminders useful in helping them to take their medications on time. SMS reminders addressed forgetfulness to take medications as prescribed, which was a commonly reported problem among study participants who had busy schedules.

Study participants were also more likely to forget taking medication on time while initiating ART as they were not yet used to the medication-taking routine:

It was actually in November I had totally forgotten and was on my bed about to fall asleep and I received an SMS on my phone and I got out of bed quickly looked for water and swallowed.

Triggered SMS arm, female, study participant

Some participants thought the SMS notifications sent to their social supporters after missed doses were useful. These participants felt motivated to take their medication after social supporters had contacted them to find out why they had not taken their medication on time:

I also like it [SMS notification] because when I have many people reminding me it gives great strength. My sister calls me when she receives an SMS reminder and asks why I didn’t swallow.

Triggered SMS arm, female, study participant

There were some cases of improved relationships between participants and social supporters as a result of participating in the study. These improvements were manifested as more frequent phones calls and in-person visits and expansion of social support networks to include friends of the social supporters. One study participant stated:

It [our relationship] has also greatly improved because now she calls me many times to remind me to swallow my pills and sometimes she visits me. Asks me how am using my device...

Triggered SMS arm, female, study participant

Social supporters also liked SMS notifications because they reminded them to support the participants in taking their medications. The SMS notifications were especially helpful for participants who often forgot to take their medication or who did not like taking the medication. Receiving SMS notifications was perceived as another helping hand to make participants more committed to taking their medication on time. One social supporter stated:

I like the fact that they [SMS notifications] remind us to remind her about her medications. Because I know that by the time I get this message, she has not opened that bottle and I need to find out why and address it. It’s her character that she naturally dreads taking medications and having these reminders and someone to remind her is very important and a very good thought from your end...I know it has helped a lot especially knowing that someone else will be told if she doesn’t take her medications on time...She doesn’t want to disappoint us even after we have united to help her in any possible way.

Female, social supporter, study participant’s sister

In cases of insufficient resources, however, social supporters could not always help participants take their medicine even after receiving the SMS notifications:

The only problem was that he finished his pills and did not have transport back...I could have sent him transport but that time I was so broke, the landlord was on my case, I did not have even enough food in the house.

Female, social supporter—study participant’s wife

#### Effort Expectancy or Peceived Ease of Use

Following study participants’ initial orientation to using the electronic adherence monitor, they found it easy to use in taking their medication:

It was very easy in opening it, moving with it, it was comfortable. It was easy to carry it. Charging it was very easy too. It would contain pills to push me for some good time...

Control arm, female, study participant

However, some participants expressed concerns about the long time required for the solar charger to fully charge the battery, especially in periods of rain when little sun was available. Some required additional training on how exactly to charge the device, whereas others were concerned about the inability of the adherence monitor to show battery levels, which created difficulties in knowing when to charge it. One participant stated:

The only complaint I have about this device is its inability to show its battery charging levels. I cannot tell whether the battery is full or empty.

Triggered SMS arm, male, study participant

Study participants reported no challenges in reading and responding to SMS reminders. Some particularly liked receiving messages in their local language (80% of reminders were in Runyankole, whereas 20% were in English):

I thought the SMS reminder would be in English so I wouldn’t understand them but they came in my local language so I felt happy and I can’t forget this SMS reminder.

Scheduled SMS arm, female study participant

Social supporters found it easy to act upon SMS notifications if they were related to participants, lived with participants or near participants’ homes or workplace, or had some tangible support to provide. One social supporter stated:

There are times when she asks for some support but I fail to help her...Like in giving her good food. When a person is sick, they need to feed well. There are also some tasks that are tiring like fetching water as we get it from far.

I know she would want to get the water brought closer to her but I sometimes cannot help her. I would need to fetch the water for her or get her someone to help her fetch the water but it is expensive to pay for this. So there are such things that you know she needs help with but I am not able to help.

Male, social supporter—study participant’s husband

#### Social Norms: HIV Status Disclosure and Stigma

Study participants stated that use of the monitoring device influenced disclosure of their HIV status to the community. For some, the adherence monitor assisted with disclosure that would potentially generate social support to help them cope with having HIV.

The monitor, especially its blinking, attracted people’s attention, which became the basis for disclosing HIV status. One study participant stated:

They saw it blinking and asked me what it was...I told them it is a bottle where I keep my medicine. And when they asked about which medicine, I told them that I was HIV positive...I thought it was not wise for me to hide my HIV status from my relatives since they had seen my bottle and had also seen me taking the medicine. I might get sick and ask them to help me get the medicine from the bottle. When they do not know the use of the bottle, they will say that we asked you what the bottle was for and you ignored us so why are you bothering us now.

Scheduled SMS arm, female, study participant

However, some study participants were uncomfortable traveling with the monitor or keeping it where other people could see it, for fear of HIV status disclosure, which resulted in stigma and discrimination:

I had gone to the village and hadn’t gone with it [device] because I didn’t want people in my village to see it. Thieves broke in my house and stole everything including the device. Later my things were retrieved and people opened it and saw that there were pills for ART so they got surprised and got to know that I was positiveI got ashamed and got it [device] from them but of course some keep talking about me and some felt sorry for me but I just left them had nothing to do for them.

Triggered SMS arm, male, study participant

Some participants also reported concerns about potential unintended HIV status disclosure through other people seeing the SMS reminders. To address these concerns, participants preferred keeping their SMS reminders private; they liked SMS reminders that would not directly link them to HIV. Greeting-related SMS reminders were preferred to general SMS messages as they would not easily raise concern if seen by someone else. One study participant stated the following:

I decided on a message [“wasiboota” or how was your day] that would not easily connect me to the clinic and my HIV status. Even when you are with people and this message comes, you do not even try to hide the message because someone who sees the message will straight away know someone who cares about you is just greeting you. But if you received a message reminder like “mira emibaazi yawe” (meaning take your medicines), someone might ask you which medicines you are going to take and there, they might know that you are sick or start asking you all sorts of questions.

Triggered SMS arm, male, study participant

Even with indirect SMS reminders, participants cited instances where regularly receiving such reminders raised some concern:

I had gone to visit at a friend's place and I had spent there like a week. Remember I go with my bottle hiding it such that he does not see it. But he read it and said, “[name], who is this person who is always sending you this kind of message ['obutumwa bwawe' meaning 'this is your message']?” I told him it is my sister who is always greeting me, but I could tell he was not convinced...

Scheduled SMS arm, female, study participant

To avoid possibilities of HIV status disclosure and its associated stigma, participants preferred having SMS notifications sent to social supporters who they trusted:

I choose my husband because other people normally talk about other peoples’ HIV status if they get to know it...If you send the messages to my husband, he reads them and tells me. But other people might start telling the whole village how I am sick and I do not like it. That is why I chose only my husband because he keeps my HIV status a secret.

Scheduled SMS arm, female, study participant

Social supporters who lived in the same home with participants were considered to be more helpful in keeping their secrets than those that stayed elsewhere and in reminding participants to take their medications:

It is good when you are staying with the person and not like neighbors or people from far. You know someone that stays with you is more likely to be very helpful in reminding you to take your medicine. They also keep your secrets well compared to someone who stays far. At times when they [outsiders] get the message, they might be tempted to talk about you or not even remind you as expected...

Scheduled SMS arm, female, study participant

#### Facilitating Conditions

Participants generally liked the appearance and form of the adherence monitor, which motivated them to use it. Specifically, they liked the monitor’s small, portable size that accommodated all of their pills, compared with the standard clinic pill bottles that necessitated participants to carry more than one bottle when traveling. They also liked the monitor’s black color, cell phone–like shape, and absence of HIV-related labels that could link them to HIV. Additionally, study participants stated that monitor’s hard outer and inside covers kept drugs safe and clean and did not make noise compared with the standard clinic pill bottles:

The white one [bottle] makes noise when one is removing the pills but this one doesn’t make noise...

Scheduled SMS arm, male, study participant

This device keeps my HIV status private and there is nothing written on it compared to the bottles I pick from the clinic...

Triggered SMS arm, female, study participant

Conditions that facilitated the use of the intervention include the monitor’s extended battery life and the availability of mobile network. One study participant stated the following:

The network was always ok. There is network here so I haven’t found any challenge with it.

Control arm, female, study participant

Supplementing solar chargers with electric chargers facilitated the use of the intervention for those with access to electrical outlets, because electric chargers enabled charging the adherence monitor indoors, thus reducing the possibility of unintended HIV status disclosure. One study participant said:

There is when you brought me an electric charger I was so happy because I stay in very busy place so charging using solar would have caused them to suspect something or even steal the solar panel. So I thank you for that.

Triggered SMS arm, female, study participant

Participants also reported several conditions that hindered the use of the SMS reminders. For example, sharing cell phones with others hampered the intervention, even though personal cell phone ownership was an inclusion criterion for the study. One study participant stated:

He [my husband] has been with the phone like for 2 weeks. So I have not seen the messages that have been sent to my phone during the time my husband had the phone.

Scheduled SMS arm, female, study participant

Additionally, some cases of phone malfunctioning constrained the receipt of some SMS reminders, and concerns were raised about not receiving SMS reminders when the phones were off or not charged or in cases of lost phones:

Yes [I received the SMS reminder] once. Just one message. That was before I lost my phone.

Triggered SMS arm, male, study participant

Unanticipated use of the technology limited the feasibility of the intervention in some cases. Due to lack of electricity, some participants allowed others to use the solar charger for their personal use, instead of keeping it to charge the adherence monitor:

My wife insisted that she needed to use it [the charger] for her phone and light. We had no power at home and she was convinced the torch would help her while she wakes up to breastfeed the baby. I decided to leave it...

Triggered SMS arm, male, study participant

### Intervention Feasibility

Data transmission generally worked well with 89% of data transmitted after a delay of 0 to 5 min. Nine percent of the device-opening data was transmitted after device signal delays of ≥60 min (because of unreliable network), which resulted in unnecessary transmission of SMS reminders. Other feasibility issues are summarized in Table 2.

Additionally, qualitative interviews revealed the following data on intervention feasibility:

#### Using the Monitor Pills Other Than Antiretroviral Therapy

There was an incidence where a participant used the adherence monitor for taking a prophylactic antibiotic (septrin or trimethoprim-sulfamethoxazole) rather than the intended ART. Using the monitor for other pills in this way interferes with the feasibility of the intervention, as use of ART was assumed by study staff. One study participant stated:

From the container [device], they [septrin] were in the paper and when ARVs got finished I put septrin in the device...

Triggered SMS arm, interview 2, male, study participant

**Table 2:** Technical feasibility of the electronic adherence monitor.

Issue	Comments
Device malfunction	3 (2%) out of 63 devices malfunctioned and were replaced: one of the devices was damaged by the participant, while the remaining had technical faults.
Data loss	191.6 (3%, 191.6/8365) of data were lost because of technical issues with the adherence monitors.
Device battery changes	Although all study participants had a solar charger and 13 had electric chargers, study staff completed 22 battery changes because of (1) poor mobile network that resulted in repeated attempts to transmit the data, which depleted the battery before its anticipated charging time; and/or (2) inability or failure of the participants to charge the batteries as requested.
Lost to follow-up	One participant was lost to follow-up because of a nonfunctioning phone number.
Change of phone numbers	Five participants changed their phone numbers
SMS^a^ reminders not sent	44 (1%) of SMS reminders were not sent because of technical challenges such as poor network coverage
Number of messages sent unnecessarily	1935 (36%) SMS reminders were sent unnecessarily. In these cases, the electronic adherence monitor was opened to take medication, but poor mobile network coverage resulted in a delay or absence of the signals.

^a^SMS: short message service.

#### Taking Pills From Another Source

Some participants at times took pills from a different source rather than the real-time adherence monitor (ie, they put multiple pills in their pocket or used another bottle for later dosing), again limiting the feasibility of the intervention. In most cases, participants were attempting to avoid possibilities of unintended HIV status disclosure, especially when traveling:

I went to Kampala and spent there a few days but I did not take the device. I just packed a few pills and left with them...I went home to find my wife had already packed the drugs for me. She put in an envelope and then folded it to fit in my trouser pockets...Yes, I do not like moving with any luggage and then if the device would fit in my trouser, I could go with it but it cannot even fit there well because my trouser would bulge. And again if I took it, everyone would see it and maybe open it to realize that I am sick and taking my medicine...

Triggered SMS arm, male, study participant

#### Opening the Real-Time Adherence Monitor Without Taking Pills

Some participants reported that their monitors were opened by other people. One opening was the result of a participant’s child’s inquisitiveness, whereas another participant’s social supporter would routinely open the monitor to verify the participant’s claim of having taken the pill. One study participant stated:

There are times when it [SMS reminder] comes after I have taken my drugs and even gone to sleep. Those are the times when [name of social supporter] tells me that he has have received the message and he asks me whether I have taken my drugs and I tell him that I have...He goes to check on the device to see if the drugs are there to confirm that I have taken...

Triggered SMS arm, interview 1, female, study participant

## Discussion

### Principal Findings

Drawing from the UTAUT model, we found that a real-time adherence monitoring intervention linked to SMS reminders and notifications is largely acceptable and feasible for supporting ART medication in rural southwestern Uganda. Overall, the key factor for acceptability appeared to be perceived usefulness; although the electronic adherence monitor was only initially intended to monitor adherence, it was also beneficial in creating a sense of being “cared for” and a sense of fear of “being caught” not adhering, both of which inspired participants to take medications to maintain their ongoing relationships with the study staff. SMS text messages not only reminded patients to take medication in time but also enabled social supporters to provide medication-taking-related support. Reminding participants to take medication was important given that participants were newly initiating ART and were likely unfamiliar with the required regularities of taking this medication. Reminders significantly improved study participants’ medication adherence [17], which helped some develop a habit of medication adherence [22]. SMS text messages have previously been reported to be acceptable among youth living with HIV in central Uganda [7], although this acceptability did not translate into improved medication adherence [27].

SMS notifications to social supporters were also perceived as useful; for example, they triggered social support and improved relationships between social supporters and patients. However, many did not result in participants getting medication-taking assistance. As reported previously, social supporters who were in good relationships with study participants, had enough resources, and lived with or near study participants were more helpful [24]. Training social supporters about the importance of medication adherence, as well as orienting them on how to assist participants with taking their medications, may improve the acceptability and feasibility of social support notifications.

Although acceptability was largely high, concerns about possibilities of HIV status disclosure and stigma or discrimination led to nonuse of the monitor by some participants who opted to take medication from different sources other than the monitor.

Concerns of monitor-related unwanted disclosure have been previously reported in China [2], whereas HIV status disclosure resulting from other people accessing SMS reminders have been reported by youth in Uganda [7]. Privacy and confidentiality remain key issues that can affect the acceptability of electronic adherence monitors [28]. In Uganda, this is accelarated by the prevailing stigma, discrimination, negative attitudes, and mistreatment of PLWHA [29]. HIV-related stigma is known to negatively impact medication adherence as it can limit social support and coping strategies [30]. Measures undertaken by our study to reduce the possibility of unintended HIV status disclosure included using SMS reminder text messages that could not easily be linked to HIV, emphasizing personal phone ownership rather than shared phones, and suggesting that study participants select social supporters who they trust and who already knew the participant’s HIV status. It is worth noting that intervention-facilitated disclosure was not entirely negative. For example, some participants who had challenges disclosing their status used the adherence monitor to disclose their HIV status to their potential social supporters.

Our results generally indicate that the intervention is technically feasible in Uganda as malfunctioning was rare. Data were obtained from most participants as expected most of the time. The fact that all participants per enrollment criteria owned personal phones, had the ability to read SMS text message reminders, and had reliable mobile network may have been contributory. The widespread adoption of cell phones and the adequate mobile network in most parts of Uganda [31] facilitates feasibility of mHealth interventions.

By leveraging the existing mobile phone infrastructure, this intervention can potentially overcome some of the barriers to medication adherence, while at the same time empowering patients to take active roles in their own health. However, variations among different populations may influence feasibility. In a survey of phone ownership in Africa, 93% of Ugandans with at least a secondary education own a cell phone, compared with 61% of those with less education [5]. In our study, 63% (123/195) of the potential participants screened had personal cell phones with adequate mobile networks, while 97% (61/63) could read English or the local language (Runyankole). Wider implementation of this intervention in less educated or resourced populations may yield differing feasibility results.

Despite the general feasibility of the intervention, challenges with access to electricity, lack of technical function when the devices are not charged, inability to charge the monitor, and the monitor’s inability to show battery level could limit the impact of the intervention. The reported use of the solar panel to provide light for the family instead of charging the monitor shows the complexity of introducing electronic monitors in resource-limited settings. In Uganda, many people in rural areas still lack access to electricity [32].

Other challenges included shared phone ownership, as well as the potential for disclosure or theft with the solar chargers that had to be charged from outside the house. Providing more training on charging the monitor, incorporating a component of showing battery levels on the monitor, and use of solar chargers could help users promptly comply with its charging demands.

Although reported by a minority of participants, monitor openings because of curiosity and use of the device for other medications could result in misclassification of adherence. Some of this bias can be reduced in analyzing the adherence data (eg, censoring more openings than would be expected per day).

Additionally, complementing adherence monitoring with assessment of biological indicators (such as viral loads), as done in this study [17], can improve the interpretation of adherence reports generated by using the monitor.

### Strengths

This analysis has a number of strengths. First, it is grounded in a well-established theory of technology acceptance—the UTAUT model. Second, it is an in-depth qualitative investigation of the experiences of study participants and their social supporters based on the state-of-art adherence measurement technology. Third, the study was conducted in a prototypical rural African setting, which has implications for similar settings, although cultural differences may have an impact on acceptability.

### Limitations

However, results may have limited generalizability as they are based on a small pilot study of 63 participants over 9 months of follow-up. It is not clear how they manifest in larger, diverse contexts, with long-term follow-up. Importantly, HIV requires lifelong treatment.

### Implications

A number of important implications for further use and development of this type of intervention arise from this study. First, the possibility of dependence on the intervention and its potential consequences on adherence after the withdrawal of the intervention warrants further investigation. We are currently carrying out a follow-up study to explore how these participants feel about the lack of the intervention and how they adhere to their ART after withdrawing the intervention.

Second, concerns of unwanted HIV status disclosure could potentially be further minimized by extending the use of the monitor in other diseases (especially nonstigmatized conditions such as hypertension), so that it is not associated with HIV. Other means for protecting against disclosure include using password-protected phones and training social supporters about the need to maintain patients’ privacy.

Additionally, although costing was not addressed in this study, it is clearly important, especially in resource-limited settings. Each real-time adherence monitor costs about US $130, which is expensive for most low-resource countries. This cost may be reduced if the adherence monitors are developed and maintained by local capacity and produced in large quantities. A recent cost-effectiveness analysis indicated that this type of monitoring would be cost-effective at <US $50, thus marking a target for device development [33].

### Conclusion

In conclusion, we found that real-time adherence monitoring, SMS text message reminders, and notifications to support ART medication adherence were largely acceptable based primarily on perceived utility and feasible in a research context within a low-resourced setting. Future efforts should focus on optimized device design, user training to overcome the challenges we encountered, cost-effectiveness studies, as well as studying the monitoring aspect of the device without accompanying interventions.

## Abbreviations

ART: antiretroviral therapy
GPRS: General Packet Radio Service
MRRH: Mbarara Regional Referral Hospital
PLWHA: people living with HIV or AIDS
RCT: randomized controlled trial
SMS: short message service
UTAUT: unified theory of acceptance and use of technology

## Appendix

Multimedia Appendix 1 CONSORT‐EHEALTH checklist (V 1.6.1).
