# Supplementary material for: Acceptability and Feasibility of Real-Time Antiretroviral Therapy Adherence Interventions in Rural Uganda: Mixed-Method Pilot Randomized Controlled Trial
Source: JMIR Mhealth Uhealth. 2018 May 17;6(5):e122. doi: 10.2196/mhealth.9031 (PMC5981052; doi:10.2196/mhealth.9031)
Supplement: Multimedia Appendix 1 [file mhealth_v6i5e122_app1.pdf]

CONSORT-EHEALTH Checklist V1.6.2 Report	Manuscript Number	9031
**(based on CONSORT-EHEALTH V1.6), available at [http://tinyurl.com/consort-ehealth-v1-6].**		
**Date completed**		
5/11/2018 11:55:14		
**by**		
Angella Musiimenta		
"Acceptability and Feasibility of Real-Time Antiretroviral Therapy Adherence Interventions in Rural Uganda: Mixed-Method Pilot Randomized Controlled Trial"		
TITLE		
**1a-i) Identify the mode of delivery in the title**		
Realtime adherence monitors, SMS Reminders and SMS notifications		
**1a-ii) Non-web-based components or important co-interventions in title**		
none		
**1a-iii) Primary condition or target group in the title**		
HIV Patients		
ABSTRACT		
**1b-i) Key features/functionalities/components of the intervention and comparator in the METHODS section of the ABSTRACT**		
Individuals living with HIV who were initiating ART were enrolled in a pilot randomized controlled trial and followed up for 9 months. The unified theory of acceptance and use of technology model, was used to<br>analyze qualitative data.		
**1b-ii) Level of human involvement in the METHODS section of the ABSTRACT**		
Study participants and social supporters participated in qualitative semistructured in-depth interviews on acceptability and feasibility of this technology guided by the research Assistants		
**1b-iii) Open vs. closed, web-based (self-assessment) vs. face-to-face assessments in the METHODS section of the ABSTRACT**		
Face to face recruitment from Mbarara Immune Suppression Syndrome(ISS) Clinic Mbarara, Mbarara District, Uganda		
**1b-iv) RESULTS section in abstract must contain use data**		
A total of 63 participants participated in the study. Participants reported that real-time monitoring intervention linked to SMS reminders and notifications are generally acceptable; the predominant feedback was perceived utility—the intervention was beneficial in motivating and reminding patients to take medication, as well as enabling provision of social support. The intervention was found to be technically feasible, as data were obtained from most participants as expected most of the time. Potential challenges included the impact of the technology on confidentiality, shared phone ownership, usability skills, and<br>availability of electricity.		
**1b-v) CONCLUSIONS/DISCUSSION in abstract for negative trials**		
n/a		
INTRODUCTION		
**2a-i) Problem and the type of system/solution**		
HIV or AIDS remains one of the biggest public health challenges, especially in developing countries, which accounts for over 70% of the 36.9 million people living with HIV or AIDS (PLWHA) globally. Despite simplification of HIV treatment (eg, single tablet and once daily dosing regimens) and improved access to ART, adherence remains challenging [1]. Nonadherence can result in HIV viremia, ART failure, and drug resistance, which can lead to deaths because of limited or complete inaccessibility of alternative therapies in resource-limited countries. Traditional approaches to adherence monitoring (eg, self-report, pill counts,<br>and pharmacy refills) do not enable real-time interventions, as they may not detect non adherence until viral suppression has been lost.		
**2a-ii) Scientific background, rationale: What is known about the (type of) system**		

mHealth technologies can potentially improve adherence to long-term medications through real-time medication and pill refill reminders, prompting social support and enabling medication monitoring [3]. Real-time wireless adherence monitors, for example, can detect adherence lapses as they occur, and interventions such as SMS reminders can be instituted before the loss of viral suppression [4]. The acceptability and feasibility of SMS reminders in Uganda, however, have not been well studied. Given the promise of real-time adherence monitoring and mobile-based interventions, the variability in effectiveness, and scarcity of literature, more thorough assessments of their acceptability and feasibility are needed.		
**Does your paper address CONSORT subitem 2b?**		
The aim of this study was to assess the acceptability and feasibility of real-time adherence monitoring linked to text messaging (short message service, SMS) reminders and notifications to support adherence among individuals living with HIV who are taking ART in rural southwestern Uganda.		
METHODS		
**3a) CONSORT: Description of trial design (such as parallel, factorial) including allocation ratio**		
All study participants received a real-time adherence monitor (Wisepill Technologies, Cape Town, South Africa; see below) and training on its function and use (eg, filling and removing antiretroviral medications and device charging). Before enrollment in the study, potential participants were assessed for adequate cellular reception in their homes on a network supported by the technology used in this study (MTN or Airtel). Participants were given solar chargers and sent an SMS to charge the monitor as needed. A simple random number generator was used to determine study arm assignments. After screening and consenting, participants were randomized 1:1:1 as follows:<br>1. Scheduled SMS (also known as SMS reminders) plus real-time adherence monitoring (scheduled SMS arm)—Study participants received an SMS reminder daily for 1 month, then weekly for 2 months. For the next 6 months, study participants received an SMS only if no signal was received from the monitor within 2 hours of the expected dosing time, and an SMS notification was sent to one to two social supporters if no signal was received for more than 48 hours.<br>2. Triggered SMS (also known as SMS reminders) plus real-time adherence monitoring (triggered SMS arm)—For the entire 9-month study period, study participants received an SMS only if no signal was received from the monitor within 2 hours of the expected dosing time. For the latter 6 of the 9 months, an SMS notification was sent to one to two social supporters if no signal was received for >48 hours.<br>3. Real-time adherence monitoring only (called the control)—Study participants in this arm received no SMS reminders.		
**3b) CONSORT: Important changes to methods after trial commencement (such as eligibility criteria), with reasons**		
n/a		
**3b-i) Bug fixes, Downtimes, Content Changes**		
n/a		
**4a) CONSORT: Eligibility criteria for participants**		

Inclusion Criteria for Patients<br>Age ≥18 years<br>• Personal cell phone ownership<br>• Ability to read short message service (SMS) messages<br>• Availability of mobile network at participants’ homes<br>• Willingness to receive SMS reminders<br>• Ability and willingness to provide informed consent<br>• Living within 20 km from Mbarara Regional Referral Hospital (to facilitate participant follow-up)<br>• Ability to identify at least one social supporter to join the study<br><br>Inclusion Criteria for Social Supporters<br>Age ≥18 years<br>• Ongoing relationships<br>• Cell phone ownership<br>• Knowledge of the study participant’s HIV status<br>• Willingness to provide informed consent<br>• History of providing social support (eg, assistance to travel to the clinic and medication adherence advice) to the study participant		
**4a-i) Computer / Internet literacy**		
n/a		
**4a-ii) Open vs. closed, web-based vs. face-to-face assessments:**		
Face to face recruitment of participants from Mbarara Immune Suppression Syndrome(ISS) Clinic Mbarara, Mbarara District, Uganda		
**4a-iii) Information giving during recruitment**		
Participants provided signed informed consent before study participation. As a cultural practice in Uganda, participants were given 10,000 Ugandan Shillings (per trip; equivalent of approximately US $4) to cover transportation costs if they came to the research offices for an interview).		
**4b) CONSORT: Settings and locations where the data were collected**		
This study involved two types of participants: PLWHA (called study participants) and their social supporters. Study participants initiating ART were recruited from the Immune Suppression Syndrome Clinic at Mbarara Regional Referral Hospital (MRRH), a rural public hospital that dispenses free ART to over 10,000 people living with HIV in southwestern Uganda. We focused on ART initiators because they are not yet accustomed to taking medication; intervening at this level could potentially result in developing medication adherence habits. HIV status<br>was identified by checking participants’ medical records.		
**4b-i) Report if outcomes were (self-)assessed through online questionnaires**		
n/a		
**4b-ii) Report how institutional affiliations are displayed**		
Massachusetts General Hospital<br>Mbarara University of Science and Technology		
**5) CONSORT: Describe the interventions for each group with sufficient details to allow replication, including how and when they were actually administered**		
**5-i) Mention names, credential, affiliations of the developers, sponsors, and owners**		
Wisepill technologies, Cape Town, South Africa<br>Dimagi<br>Yo Soultions<br>Investigators<br>Principal Investigator: Jessica Haberer, MD, MS Massachusetts General Hospital<br>Principal Investigator: Angella Musiimenta, PhD Mbarara University of Science and Technology<br><br>Sponsors and Collaborators<br>Massachusetts General Hospital<br>Mbarara University of Science and Technology		
**5-ii) Describe the history/development process**		
**5-iii) Revisions and updating**		

no major revisions and updates were done		
**5-iv) Quality assurance methods**		
Following each interview, the research investigators reviewed transcripts for quality, clarity, and detail.		
**5-v) Ensure replicability by publishing the source code, and/or providing screenshots/screen-capture video, and/or providing flowcharts of the algorithms used**		
n/a		
**5-vi) Digital preservation**		
n/a		
**5-vii) Access**		
All study participants received a real-time adherence monitor (Wisepill Technologies, Cape Town, South Africa; see below) and training on its function and use (eg, filling and removing antiretroviral medications and device charging). Before enrollment in the study, potential participants were assessed for adequate cellular reception in their homes on a network supported by the technology used in this study (MTN or Airtel). Participants were given solar chargers and sent an SMS to charge the monitor as needed. A simple random number generator was used to determine study arm assignments.		
**5-viii) Mode of delivery, features/functionalities/components of the intervention and comparator, and the theoretical framework**		
After screening and consenting, participants were randomized 1:1:1 as follows:<br>1. Scheduled SMS (also known as SMS reminders) plus real-time adherence monitoring (scheduled SMS arm)—Study participants received an SMS reminder daily for 1 month, then weekly for 2 months. For the next 6 months, study participants received an SMS only if no signal was received from the monitor within 2 hours of the expected dosing time, and an SMS notification was sent to<br>one to two social supporters if no signal was received for more than 48 hours.<br>2. Triggered SMS (also known as SMS reminders) plus real-time adherence monitoring (triggered SMS arm)—For the entire 9-month study period, study participants received an SMS only if no signal was received from the monitor<br>within 2 hours of the expected dosing time. For the latter 6 of the 9 months, an SMS notification was sent to one to two social supporters if no signal was received for >48 hours.<br>3. Real-time adherence monitoring only (called the control)—Study participants in this arm received no SMS reminders.<br>The Unified Theory of acceptance and use of Technology model guided the design of the data collection tools collection and analysis of data.		
**5-ix) Describe use parameters**		
Device: Fixed SMS, real-time monitoring<br>Device: Triggered SMS, real-time monitoring		
**5-x) Clarify the level of human involvement**		
Research assistants:<br>-training the participants on using the devices<br>-Replacement of the malfunctioning devices<br>-Data Collection<br>-Interview transcription		
**5-xi) Report any prompts/reminders used**		
Text messaging (short message service, SMS) reminders and notifications to support adherence among individuals living with HIV who are taking ART in rural southwestern Uganda.		
**5-xii) Describe any co-interventions (incl. training/support)**		
All study participants were trained to use the realtime adherence monitors (Filling and removing medication and device charging)		
**6a) CONSORT: Completely defined pre-specified primary and secondary outcome measures, including how and when they were assessed**		
Antiretroviral Therapy (ART) Adherence Levels [Time Frame: real time (for 9 months)] ART adherence in each study arms. Adherence was measured by the Wisepill real-time adherence monitor and calculated as the number of monitor opening signals received divided by the number of monitor opening signals expected, capped at 100%.		
**6a-i) Online questionnaires: describe if they were validated for online use and apply CHERRIES items to describe how the questionnaires were designed/deployed**		
We never used online questionnaires		
**6a-ii) Describe whether and how "use" (including intensity of use/dosage) was defined/measured/monitored**		

Powered by a rechargeable battery, the real-time adherence monitor (Figure 1) is a medication container that can hold up to sixty small pills. When an individual opens it to take pills, the device records a date-and-time stamp. An internal modem and subscriber identity module card enable the device to send a real-time mobile signal to a secure Web server (hosted in South Africa) by General Packet Radio Service (GPRS). Receipt of this signal was taken as a proxy for taking medication. GPRS maintains the data in transit until acknowledgment of receipt by the Web server, which minimizes possible data loss because of power failure or lack of Internet connectivity. Data transmission is backed up by the SMS to mitigate possible temporal GPRS network disconnections. In the event of inadequate mobile network coverage, the monitor stores openings in flash memory and sends them when the network becomes available. The monitor also transmits a daily heart beat that indicates current battery life, remaining airtime balance, and signal strength as indication of its functionality. The monitor can be charged using electricity or a solar device. Its battery life was 3 months at the time of the study but has since been improved to 6 months.		
**6a-iii) Describe whether, how, and when qualitative feedback from participants was obtained**		
Semistructured qualitative interviews were conducted after month 3 (known as interview 1) and after the first 48-hour lapse (known as interview 2), or at study exit if there was no such lapse (also known as interview 2), reflecting two planned interviews per participant. In-depth semistructured interviews with a purposeful sample of social supporters were conducted within 2 weeks of a lapse by their respective study participant. Their selection was based on the study participant's explanation for the lapse, social support characteristics, and variations in the types of social support provided. Closed and open-ended questions were asked of social supporters at exit exploring various aspects such as challenges and experiences to social support and understanding of and responses to the intervention SMS notifications and the type of voluntary and requested help or support presently given to the study participant toward adherence.		
**6b) CONSORT: Any changes to trial outcomes after the trial commenced, with reasons**		
This study involved two types of participants: PLWHA (called study participants) and their social supporters. Study participants initiating ART were recruited from the Immune Suppression Syndrome Clinic at Mbarara Regional Referral Hospital (MRRH), a rural public hospital that dispenses free ART to over 10,000 people living with HIV in southwestern Uganda. We focused on ART initiators because they are not yet accustomed to taking medication; intervening at this level could potentially result in developing medication adherence habits. HIV status was identified by checking participants' medical records.		
**7a) CONSORT: How sample size was determined**		
**7a-i) Describe whether and how expected attrition was taken into account when calculating the sample size**		
**7b) CONSORT: When applicable, explanation of any interim analyses and stopping guidelines**		
Antiretroviral Therapy (ART) Adherence Levels [Time Frame: real time (for 9 months)] ART adherence in each study arms. Adherence was measured by the Wisepill real-time adherence monitor and calculated as the number of monitor opening signals received divided by the number of monitor opening signals expected, capped at 100%.		

**8a) CONSORT: Method used to generate the random allocation sequence**		
Randomized using 1:1:1 ration into three arms		
**8b) CONSORT: Type of randomisation; details of any restriction (such as blocking and block size)**		
Participants received a real-time adherence monitor and were randomized to one of the following study arms: (1) scheduled SMS, (2) SMS triggered by missed or delayed doses, or (3) no SMS.		
**9) CONSORT: Mechanism used to implement the random allocation sequence (such as sequentially numbered containers), describing any steps taken to conceal the sequence until interventions were assigned**		
Participants received a real-time adherence monitor and were randomized to one of the following study arms: (1) scheduled SMS, (2) SMS triggered by missed or delayed doses, or (3) no SMS.		
**10) CONSORT: Who generated the random allocation sequence, who enrolled participants, and who assigned participants to interventions**		
The research Assistants at the clinic generated the sequence, enrolled the participants and allocated the participants to the different intervention arms		
**11a) CONSORT: Blinding - If done, who was blinded after assignment to interventions (for example, participants, care providers, those assessing outcomes) and how**		
**11a-i) Specify who was blinded, and who wasn't**		
participants were not blinded		
**11a-ii) Discuss e.g., whether participants knew which intervention was the "intervention of interest" and which one was the "comparator"**		
The participants were choosen randomly to different study arms		
**11b) CONSORT: If relevant, description of the similarity of interventions**		
n/a		
**12a) CONSORT: Statistical methods used to compare groups for primary and secondary outcomes**		
n/a		
**12a-i) Imputation techniques to deal with attrition / missing values**		
n/a		
**12b) CONSORT: Methods for additional analyses, such as subgroup analyses and adjusted analyses**		

The unified theory of acceptance and use of technology (UTAUT) model, which has been shown to predict a substantial portion of the acceptance of health information technology, served as the conceptual framework for this analysis [25]. In this model, technology adoption is influenced by four major constructs as perceived by an individual user: (1) performance expectancy or perceived usefulness, (2) effort expectancy or percieved ease of use, (3) social norms (ie, how others perceive the individual's use of the intervention), and (4) facilitating conditions (ie, the availability of technical and organizational infrastructure to support use of the intervention). We used an inductive, content analytic approach to analyze the qualitative data [26]. For this paper, we used the qualitative data management computer software program NVIVO 10 (QSR International., Melbourne, Australia) to organize the data. With substantial input from JEH, NCW, TW, and MAW, AM reviewed transcripts for content relevant to acceptability drawing from the UTAUT model; developed a coding scheme based on the content identified; coded the data; sorted and reviewed the coded data to develop descriptive categories; and mapped the descriptive categories onto the domains of the UTAUT model (focusing on perceived usefulness, perceived ease of use, social norms, and facilitating conditions). Illustrative citations were then selected from the coded data. Quantitative data about the feasibility of the intervention were recorded and summarized descriptively using STATA 13 (StataCorp., College Station, Texas, USA).		
RESULTS		
**13a) CONSORT: For each group, the numbers of participants who were randomly assigned, received intended treatment, and were analysed for the primary outcome**		
Of 195 screened individuals, 63 were enrolled in the study from September 2013 to October 2014, whose 9-month follow-up ended in June 2015. One participant was later discovered to be HIV negative and was excluded from the analysis.		
**13b) CONSORT: For each group, losses and exclusions after randomisation, together with reasons**		
n/a		
**13b-i) Attrition diagram**		
n/a		
**14a) CONSORT: Dates defining the periods of recruitment and follow-up**		
September 2013 to June 2015		
**14a-i) Indicate if critical "secular events" fell into the study period**		
n/a		
**14b) CONSORT: Why the trial ended or was stopped (early)**		
n/a		
**15) CONSORT: A table showing baseline demographic and clinical characteristics for each group**		
n/a		
**15-i) Report demographics associated with digital divide issues**		
n/a		
**16a) CONSORT: For each group, number of participants (denominator) included in each analysis and whether the analysis was by original assigned groups**		
**16-i) Report multiple "denominators" and provide definitions**		

Participants included in the study, n 63<br>Participants who completed the study, n (%) 58 (92)<br>Had electricity in their homes, n (%) 38 (65)<br>Females, n (%) 41 (65)<br>Able to read and write, n (%) 61 (97)<br>Median age in years 30<br>Median follow-up time in months 8.9		
**16-ii) Primary analysis should be intent-to-treat**		
n/a		
**17a) CONSORT: For each primary and secondary outcome, results for each group, and the estimated effect size and its precision (such as 95% confidence interval)**		
n/a		
**17a-i) Presentation of process outcomes such as metrics of use and intensity of use**		
n/a		
**17b) CONSORT: For binary outcomes, presentation of both absolute and relative effect sizes is recommended**		
n/a		
**18) CONSORT: Results of any other analyses performed, including subgroup analyses and adjusted analyses, distinguishing pre-specified from exploratory**		
n/a		
**18-i) Subgroup analysis of comparing only users**		
n/a		
**19) CONSORT: All important harms or unintended effects in each group**		
Study participants stated that use of the monitoring device<br>influenced disclosure of their HIV status to the community. For<br>some, the adherence monitor assisted with disclosure that would<br>potentially generate social support to help them cope with having<br>HIV. The monitor, especially its blinking, attracted people’s attention,<br>which became the basis for disclosing HIV status.		
**19-i) Include privacy breaches, technical problems**		
However, some study participants were uncomfortable traveling<br>with the monitor or keeping it where other people could see it,<br>for fear of HIV status disclosure, which resulted in stigma and<br>discrimination:<br><br>"I had gone to the village and hadn’t gone with it<br>[device] because I didn’t want people in my village<br>to see it. Thieves broke in my house and stole<br>everything including the device. Later my things were<br>retrieved and people opened it and saw that there<br>were pills for ART so they got surprised and got to<br>know that I was positiveI got ashamed and got it<br>[device] from them but of course some keep talking<br>about me and some felt sorry for me but I just left<br>them had nothing to do for them. [Triggered SMS<br>arm, male, study participant"		
**19-ii) Include qualitative feedback from participants or observations from staff/researchers**		

"I like the fact that they [SMS notifications] remind us to remind her about her medications. Because I know that by the time I get this message, she has not opened that bottle and I need to find out why and address it. It’s her character that she naturally dreads taking medications and having these reminders and someone to remind her is very important and a very good thought from your end...I know it has helped a lot especially knowing that someone else will be told if she doesn’t take her medications on time...She doesn’t want to disappoint us even after we have united to help her in any possible way. [Female, social supporter, study participant’s sister]"		
DISCUSSION		
**20) CONSORT: Trial limitations, addressing sources of potential bias, imprecision, multiplicity of analyses**		
**20-i) Typical limitations in ehealth trials**		
However, results may have limited generalizability as they are based on a small pilot study of 63 participants over 9 months of follow-up. It is not clear how they manifest in larger, diverse contexts, with long-term follow-up. Importantly, HIV requires lifelong treatment.		
**21) CONSORT: Generalisability (external validity, applicability) of the trial findings**		
**21-i) Generalizability to other populations**		
Third, the study was conducted in a prototypical rural African setting, which has implications for similar settings, although cultural differences may have an impact on acceptability.		
**21-ii) Discuss if there were elements in the RCT that would be different in a routine application setting**		
n/a		
**22) CONSORT: Interpretation consistent with results, balancing benefits and harms, and considering other relevant evidence**		
**22-i) Restate study questions and summarize the answers suggested by the data, starting with primary outcomes and process outcomes (use)**		
Drawing from the UTAUT model, we found that a real-time adherence monitoring intervention linked to SMS reminders and notifications is largely acceptable and feasible for supporting ART medication in rural southwestern Uganda. Overall, the key factor for acceptability appeared to be perceived usefulness; although the electronic adherence monitor was only initially intended to monitor adherence, it was also beneficial in creating a sense of being “cared for” and a sense of fear of “being caught” not adhering, both of which inspired participants to take medications to maintain their ongoing relationships with the study staff. SMS text messages not only reminded patients to take medication in time but also enabled social supporters to provide medication-taking-related support. Reminding participants to take medication was important given that participants were newly initiating ART and were likely unfamiliar with the required regularities of taking this medication. Reminders significantly improved study participants’ medication adherence [17], which helped some develop a habit of medication adherence		
**22-ii) Highlight unanswered new questions, suggest future research**		
Future efforts should focus on optimized device design, user training to overcome the challenges we encountered, cost-effectiveness studies, as well as studying the monitoring aspect of the device without accompanying interventions.		
Other information		
**23) CONSORT: Registration number and name of trial registry**		
NCT01957865		
**24) CONSORT: Where the full trial protocol can be accessed, if available**		
n/a		
**25) CONSORT: Sources of funding and other support (such as supply of drugs), role of funders**		
The study was funded by a grant from the US National Institutes of Health (R34MH100940).		
**X26-i) Comment on ethics committee approval**		

Ethical approvals for this study were obtained from the Institutional Review Committee of Mbarara University of Science and Technology, the Uganda National Council for Science and Technology, and the Partners Human Research Committee at Massachusetts General Hospital. Participants provided signed informed consent before study participation. All participants' data were securely stored electronically and protected by passwords. As a cultural practice in Uganda, participants were given 10,000 Ugandan Shillings (per trip; equivalent of approximately US $4) to cover transportation costs if they came to the research offices for an interview).		
**x26-ii) Outline informed consent procedures**		
Participants provided signed informed consent before study participation, where they were informed about the purpose of the study, the benefits, risks involved, voluntary participation and withdraw from the study at any time they wanted without affecting their care at the Hospital		
**X26-iii) Safety and security procedures**		
All participants' data were securely stored electronically and protected by passwords		
**X27-i) State the relation of the study team towards the system being evaluated**		
n/a		
